# Does the Microbiome Affect the Outcome of Renal Transplantation?

**DOI:** 10.3389/fcimb.2020.558644

**Published:** 2020-12-23

**Authors:** Paul M. Campbell, Gavin J. Humphreys, Angela M. Summers, Joanne E. Konkel, Christopher G. Knight, Titus Augustine, Andrew J. McBain

**Affiliations:** ^1^ School of Health Sciences, Faculty of Biology, Medicine and Health, The University of Manchester, Manchester, United Kingdom; ^2^ Department of Renal and Pancreatic Transplantation, Manchester University NHS Foundation Trust, Manchester Academic Health Science Centre, Manchester, United Kingdom; ^3^ Lydia Becker Institute of Immunology and Inflammation, Faculty of Biology, Medicine and Health, The University of Manchester, Manchester, United Kingdom; ^4^ School of Natural Sciences, Faculty of Science and Engineering, The University of Manchester, Manchester, United Kingdom

**Keywords:** oral microbiome, gut microbiome, kidney transplant, surgery, renal allograft, urinary microbiome, chronic kidney disease, end stage renal disease

## Abstract

The role of the human microbiome in health and disease is becoming increasingly apparent. Emerging evidence suggests that the microbiome is affected by solid organ transplantation. Kidney transplantation is the gold standard treatment for End-Stage Renal Disease (ESRD), the advanced stage of Chronic Kidney Disease (CKD). The question of how ESRD and transplantation affect the microbiome and *vice versa* includes how the microbiome is affected by increased concentrations of toxins such as urea and creatinine (which are elevated in ESRD), whether restoration of renal function following transplantation alters the composition of the microbiome, and the impact of lifelong administration of immunosuppressive drugs on the microbiome. Changes in microbiome composition and activity have been reported in ESRD and in therapeutic immunosuppression, but the effect on the outcome of transplantation is not well-understood. Here, we consider the current evidence that changes in kidney function and immunosuppression following transplantation influence the oral, gut, and urinary microbiomes in kidney transplant patients. The potential for changes in these microbiomes to lead to disease, systemic inflammation, or rejection of the organ itself is discussed, along with the possibility that restoration of kidney function might re-establish orthobiosis.

## Introduction

The human microbiome can confer multiple benefits to health (Wang et al., 2017). Examples include aiding development of organs (Goyal et al., 2015) and the innate and adaptive immune systems (Lee and Mazmanian, 2010; Honda and Littman, 2016; Thaiss et al., 2016) and resistance to infection (Bäumler and Sperandio, 2016).

The immune system controls the human microbiome, for example, in the small intestine, where the antimicrobial peptide RegIIIγ restricts the number of bacteria in contact with the epithelial surface, and secreted innate immune effectors can alter the composition of luminal microbiota (Vaishnava et al., 2011; Hooper et al., 2012). Current evidence suggests that the microbiome differs in diseases where immune function is altered (Belkaid and Hand, 2014; Idris et al., 2017), including: hematological malignancies (Bergmann, 1989; Galili et al., 1992; Wang et al., 2014), pro-inflammatory cancers (Pushalkar et al., 2011; Farrell et al., 2012; Han et al., 2014; Hu et al., 2015; Fan et al., 2018) and inflammatory bowel diseases (Docktor et al., 2012). The effects of microbiome change on patient outcomes have, however, not been fully elucidated. Organ transplantation and subsequent immunosuppression offer the opportunity to study the effects of immunosuppression longitudinally.

Despite advances in treatment, data yielded by the ELITE-Symphony trial reported that infections and rejection, broadly associated with over-immunosuppression and under-immunosuppression respectively, occurred in approximately 25% of cases within one year of renal transplantation (Cippà et al., 2015). A knowledge gap remains regarding optimal immunosuppression, which may be considered to be a balance of risk between the two outcomes. Understanding the processes driving microbiome change and potential downstream consequences to health could therefore inform prediction, prevention, and management of post-transplant outcomes. This review considers evidence that microbiome composition is linked to outcome in kidney transplant surgery. We additionally consider how changes in kidney function can affect both the immune system and the microbiome, and the evidence that microbiome alteration could lead to acute rejection.

## Use of Immunosuppressants in Transplantation

Advancements in immunosuppressant therapies have led to improvements in the success of kidney transplantation. Ciclosporin and tacrolimus have been used in this application since the 1980s (Spencer et al., 1997; Spolidorio et al., 2006; Colombo and Ammirati, 2011). Both calcineurin-inhibiting drugs interact with intracellular proteins of the immunophilin family; the former forms a complex with cyclophilin, and the latter with FK506-binding protein 12 with greater molar potency (Halloran, 2004). Since 90% of kidney transplant recipients in the US received a calcineurin inhibitor-based regime in 2012 (Matas et al., 2014), the majority of microbiome studies in this area are based on patient cohorts following ciclosporin or tacrolimus-based regimes. However, modern kidney transplantation procedures involve several other immunosuppressive agents, including prednisone, mycophenolate mofetil, sirolimus, and azathioprine, with or without added steroids (Diaz et al., 2013).

Immunosuppressant drugs, including tacrolimus, can function as a macrolide antibiotic and such properties are likely to affect bacteria. Moreover, prophylactic antibiotics may also significantly alter the microbiome (Jakobsson et al., 2010; Korpela et al., 2016), confounding investigations into the effect of immunosuppression. Comparisons between cohorts may be complicated by the lack of consensus on optimal perioperative prophylaxis regimes (Orlando et al., 2015; Bliven et al., 2018), but such confounders may be avoided by using living organ donors as controls paired with their recipients. However, the effect of factors which solely affect chronic kidney disease patients pre-transplant (specialized diet, uremic toxins) are less easily disentangled from immunosuppression.

## The Oral Microbiome During Immunosuppression

There is a longstanding association between immunosuppressive agents and oral disease. Gingival hyperplasia, for example, has been associated with the immunosuppressant ciclosporin, and kidney-transplantation (Rateitschak-Plüss et al., 1983) where bacteria-induced inflammation could be affected by transplant-driven microbiome changes (Brown et al., 1991). In a large study of kidney transplant patients, 60% had at least one type of oral mucosal ulcer (de la Rosa-García et al., 2005). Similar studies indicate that these lesions are common in transplant or immunocompromised cohorts with causal links to oral microbiome constituents. Oral candidiasis is also more common in transplant recipients and immunosuppressed cohorts (King et al., 1994; Olczak-Kowalczyk et al., 2010). Whether the overgrowth and increased prevalence of *Candida* spp. in this context is caused by failure of the immunocompromised host to maintain normal suppression of its growth, or a side effect of prophylactic antibiotic use (Lynch, 1994), remains to be discerned.

Immunosuppression has been reported to alter the composition of the oral microbiome beyond six months post-transplant (Diaz et al., 2013; Fricke et al., 2014). A study comparing subgingival plaque bacteria reported increased bacterial counts and clinical indicators of gingival overgrowth post-transplantation (Saraiva et al., 2006). More recently, significant and persistent differences have been reported in kidney transplant recipients before and after transplant (**Table 1**). The potential for these changes to negatively impact patient health is suggested by increases in opportunistic pathogens, which has been reported even where concurrent differences in alpha-diversity and global community structure are not observed (Diaz et al., 2013).

**Table 1:** Summary of recent studies reporting microbiome-associated differences (and their, potentially, related, post-operative effects) using kidney transplant recipient (KTR) cohorts.

Study	Microbiome Site	Immunosuppressive Agents Administered	Comparison	Microbiome Change Reported	Post-Operative Outcome Reported	Method of Detection
(Diaz et al., 2013)	Oral	Prednisone, Mycophenolate mofetil, Tacrolimus, Ciclosporin, Sirolimus, Azathioprine	Kidney and Cardiac Transplant Recipients (20) vs healthy cohort (19)	Increased prevalence of potentially opportunistic pathogens (*K. pneumoniae, P. fluorescens, Acinetobacter* spp*, Vibrio* spp.*, Enterobacteriaceae* spp.)	Cluster of opportunistic pathogens correlated with serum C-reactive protein, potential link between flora and systemic inflammation	16S rRNA sequencing
(Saraiva et al., 2006)	Oral	Ciclosporin	35 KTRs before and after procedure	Increase in total viable counts of microorganisms on day 90 after surgery	Increased gingival overgrowth Beta-hemolytic *Streptococcus* detected less frequently in gingival overgrowth	Culturing and oral disease diagnoses
(Spolidorio et al., 2006)	Oral	Ciclosporin and Tacrolimus	KTRs receiving ciclosporin (88) and tacrolimus (67)	Increased levels of *Candida* spp. detected in ciclosporin group	Increased gingival overgrowth, candida infection, squamous cell carcinoma and herpes simplex in ciclosporin group	Culturing and oral disease diagnoses
(Swarte et al., 2020)	Gut	Ciclosporin, tacrolimus, azathioprine, mycophenolate mofetil, prednisolone	KTRs after procedure (139) and healthy controls (105).	Lower Shannon diversity detected in KTR group. Use of mycophenolate mofetil correlated with lower diversity	na	16S rRNA sequencing
(Lee et al., 2019)	Gut	Anti-thymocyte globulin, basiliximab, tacrolimus, belatacept, mycophenolate mofetil, prednisone	71 KTRs (Diarrheal specimens vs non-diarrheal)	Lower Shannon diversity in diarrheal specimens. Lower relative abundance of 13 genera in diarrheal fecal specimens vs non-diarrheal	26 out of 28 diarrheal specimens negative for infectious etiologies. Diarrhea specimens predicted to have lower abundance of metabolic genes	16S rRNA sequencing and PICRUSt (Langille et al., 2013)
(Zaza et al., 2017)	Gut	Everolimus, tacrolimus, mycophenolate mofetil	9 KTRs receiving everolimus and 11 KTRs receiving tacrolimus	Alpha diversity not significantly different Three functional genes (fliNY, pilM and msrA) discriminated microbiome profile of each group	na	Taxonomic profiling *via* 16S rRNA sequencing & functional analysis using DIAMOND (Buchfink et al., 2015)
(Lee et al., 2014)	Gut	Tacrolimus and Mycophenolate acid or Mycophenolate mofetil	26 KTRs before and after procedure	Increase in relative abundance of Proteobacteria post-transplant	Post-transplant diarrhea associated with lower Shannon diversity index PCoA^1^ and LEfSe^2^ distinguish between acute rejection and no acute rejection group Fecal abundance of *Enterococcus* associated with urinary tract infection	16S rRNA sequencing
(Wu et al., 2018)	Urinary	Tacrolimus, Ciclosporin and none.	35 KTRs with Chronic Allograft Dysfunction vs 32 KTRs without	Shannon diversity index and beta diversity not significantly different between groups. 21 OTUs^3^ significantly higher in Chronic Allograft Dysfunction cases	na	16S rRNA sequencing
(Modena et al., 2017)	Urinary	Tacrolimus, Mycophenolate mofetil and Prednisone	25 KTRs developing IFTA vs 23 KTRs with normal biopsies and 20 non-transplant controls	*Streptococcus* lower in IFTA^4^ and “normal” KTR males vs healthy male controls (after 1 month). Further decreased after 6–8 months in IFTA males, but normalized in “normal” KTR males IFTA associated with a loss in dominant resident urinary microbes in males, and parallel increase in nonresident, pathogenic bacteria in males and females	na	16S rRNA sequencing
(Fricke et al., 2014)	Blood, Urinary, Oral and Rectal	Not Reported	60 KTRs before and after procedure	Differences in structure observed between pre- and 1 month post-transplant (persisted after 6 months) Decreases in *Proteobacteria Escherichia*, *Porphyromonas* (urine) and *Haemophilus*, *Neisseria*, *Pasteurella* (oral)	Pre-transplant microbiota associated with subsequent rejection and infection events	16S rRNA sequencing

^1^Principal coordinates analysis.

^2^Linear discriminant analysis effect size.

^3^Operational taxonomic units.

^4^Interstitial fibrosis and tubular atrophy.

na, not applicable.

## The Contribution of Changes in the Oral Microbiome to Transplant-Associated Disease

Increased prevalence of opportunistic pathogens in the oral microbiome of transplant patients including *Enterobacteriaceae*, *Pseudomonas fluorescens*, *Actinetobacter* spp., and *Vibrio* spp., have been reported (Diaz et al., 2013). Some of the same taxa incur greater relative abundance in critically ill patients (McDonald et al., 2016). Post-transplant infections remain the leading cause of morbidity and mortality in kidney transplantation, occurring in 31% of recipients within the first two years (Karuthu and Blumberg, 2012; Cowan et al., 2018). The source of infections within around one month of transplantation may be hositial-acquired whereas those in the subsequent five months may be due to opportunistic pathogens beginning to take advantage of immunosuppression (Karuthu and Blumberg, 2012). Oral microbiome analysis indicates that some taxa increasing in abundance following transplantation are those associated with common post-transplant infections; particularly *Klebsiella pneumoniae* and *Pseudomonas* spp. (Hlava et al., 2009; Diaz et al., 2013). Indeed, extra-oral colonization by opportunistic oral microbiota has been associated with a large number of diseases (Han and Wang, 2013), representing a significant risk to immunocompromised patients after transplantation.

Oral cancers are frequently observed in kidney transplant recipients (Regev et al., 1992; Thomas et al., 1993; Seymour et al., 1997; Yoon et al., 2003; Spolidorio et al., 2006; Campistol and Schena, 2007). Patients undergoing immunosuppression are generally more susceptible to some systemic cancers (Gardner et al., 2004; Sinha et al., 2004; Gutierrez-Dalmau and Campistol, 2007), but whether such higher rates of oral cancer are linked to altered microbial activity, such as through increased inflammation, or unconnected side-effects of the immunosuppression itself, is unclear. It has been proposed that colonization of dysplastic oral tissue by *Candida* spp. might accelerate progression towards oral squamous cell carcinoma. Elsewhere, microbe-driven inflammation by *Helicobacter pylori* and *Fusobacterium nucleatum* have been linked to carcinogenesis (Chiba et al., 2008; Castellarin et al., 2012; Kostic et al., 2012). Alongside direct action on epithelial cells, *H. pylori* indirectly drives carcinogenesis through the secretion of virulence factors (e.g. γ-Glutamyl transpeptidase) causing oxidative stress and long-term inflammation (Díaz et al., 2018). Similarly, in the oral cavity, carcinoma could involve secondary metabolite-driven inflammation, the production of genotoxic substances such as acetaldehyde, or cell invasion (Healy and Moran, 2019).

## Pre-Transplant, Co-Morbidities Exacerbate Systemic Inflammation

Evidence for the interaction between chronic kidney disease (CKD) and chronic periodontitis (CP) has been reviewed by Hickey et al. (2020). Certain bacteria are understood to cause local kidney damage, e.g. acute post-streptococcal glomerulonephritis, a common nephric condition often attributed to group A streptococci (Ahn and Ingulli, 2008). Systemic interplay between chronic periodontitis and chronic kidney disease may however go beyond this to exacerbate both conditions, with CKD-associated pH changes and gingival hyperplasia creating favorable conditions for the growth of oral pathogens (Listgarten, 1986), and CP-associated systemic inflammation aggravating that already associated with CKD (Paraskevas et al., 2008; Wahid et al., 2013).

## Salivary Urea in Kidney Disease and Transplantation

CKD causes the accumulation of waste products, including urea, to concentrate in the blood and saliva (Pandya et al., 2016). As a consequence, salivary urea concentration may be up to four times higher in CKD patients than healthy individuals (Lasisi et al., 2016). Oral bacteria including *Streptococcus salivarius* can metabolize urea to carbonic acid and ammonia, with a net increase in pH (Casiano-Colón and Marquis, 1988; Wijeyeweera and Kleinberg, 1989; Chen et al., 1996; Morou-Bermudez and Burne, 1999; Yaling et al., 2006; Nascimento et al., 2009) which may differentially affect the growth of oral bacteria with higher pH optima (Bowden and Hamilton, 1987; Quivey et al., 2000; Marsh and Devine, 2011; Ratzke and Gore, 2018), possibly contributing to differences seen in the microbiome of CKD patients versus healthy controls (Hu et al., 2018). Alkalization has been suggested to have a role in protection against acidification and demineralization of enamel (Kleinberg et al., 1982; Burne and Marquis, 2000) which may influence the lower caries incidence reported in CKD patients (Peterson et al., 1985; Al Nowaiser et al., 2003; Andrade et al., 2013). Moreover, shifts in oral pH could affect the immune system (Lardner, 2001; Erra Díaz et al., 2018).

Diseases that increase urea concentrations have also been linked to various oral co-morbidities, chronic renal failure patients may have increased dental plaque, enamel defects, and gingival enlargement compared with healthy individuals (Al Nowaiser et al., 2003). Since the objective of kidney transplantation is to restore kidney function, which normalizes urea concentrations, a better understanding of downstream consequences on the oral microbiome may inform dental care post-transplant. It is unclear whether the restoration of kidney function causes the oral microbiome to return to original function and composition and how this affects the risk of future oral disease. Also relevant would be the effect of resulting ammonia concentration change on ammonia-oxidizing archaea (Pester et al., 2011). Oral archaea, including those capable of oxidizing ammonia, have been associated with periodontal disease (Lepp et al., 2004; de Macario and Macario, 2009; Probst et al., 2013) although many studies focus solely on eubacteria.

## The Gut Microbiome Is Structurally Altered by Immunosuppressants

The large intestine is the most heavily colonized site in the body where microbial cell density exceeds all other human microbiome sites by at least two orders of magnitude (Sender et al., 2016). The gut microbiome has a profound influence on host metabolism and immunity (Sekirov et al., 2010), and its composition remains relatively stable in healthy adults (Huttenhower et al., 2012). Following transplantation, however, significant changes to structure have been reported (**Table 1**).

In solid organ transplantation, the immunosuppressants ciclosporin and tacrolimus have been well documented to result in significant structural changes to the gut microbiome. A large liver transplantation study reported that recipients, largely administered with ciclosporin or tacrolimus (plus mycophenalite mofetil), had decreased *Bifidobacterium* spp., *Lactobacillus* spp. and *Faecalibacterum prausnitzii*, and significantly higher *Enterobacteriaceae* and *Enterococcus* spp. (Wu et al., 2012). Although gut microbiomes are individualized (Dethlefsen et al., 2007) key compositional changes including lower overall diversity (Swarte et al., 2020) and increases in the relative abundance of Proteobacteria (Lee et al., 2014) have been reported post-transplantation. Whether fecal microbiota transplantation could restore the microbiome post-transplant, an effective treatment for *Clostridium difficile* infection, remains to be studied at scale (Al Khodor and Shatat, 2017).

There is some evidence that manipulating the immune response *via* changes in the gut microbiome could be used to modify allograft outcomes. This has been investigated in murine models, where treatment using gut microbiota has significantly improved skin allograft survival *via* tolerogenic immune responses (Zhang et al., 2018). Moreover, manipulating the growth of species such as *Faecalibacterium prausnitzii* which are capable of metabolizing the immunosuppressant tacrolimus might reduce the requirement to increase immunosuppressant dose later in treatment (Lee et al., 2015; Guo et al., 2019). This could reduce the prevalence and severity of side effects caused by an increased dose. Ultimately both examples highlight the high motivation for, and utility of, understanding how microbiome change could manipulate transplant outcome.

## Key Structural and Functional Changes in the Gut Could Cause Rejection

Immunosuppressants can affect the microbiome in complex and co-occurring ways. For example, the gut microbiome of patients treated with everolimus in combination with mycophenolate mofetil had similar alpha diversity to those treated with tacrolimus in combination with mycophenolate mofetil (Zaza et al., 2017). By going beyond comparisons of solely taxonomic composition, the same study reported that the relative abundance of three functional genes could distinguish between these groups. Metabolic pathways usually remain stable within healthy populations (Huttenhower et al., 2012). Here, flagellar motor switch protein (fliNY) and type IV pilus assembly protein pilM (pilM) genes were found to be enriched in tacrolimus-treated patients, whereas macrolide transport system msrA (msrA) was more abundant in the everolimus group.

Fecal samples from kidney transplant patients with post-transplant diarrhea had lower microbial diversity and abundance of 13 commensal genera (Lee et al., 2019). Whereas patients without diarrhea had significantly lower relative abundances of 3 genera: *Enterococcus*, *Escherichia*, and *Lachnoclostridium*. Significant differences were also reported in several metabolic pathways in diarrheal groups, including decreases in metabolic pathways involved in sucrose, starch, and amino acid metabolism. The most significant change was a reduction in cellobiose phosphorylase, a gene involved in cellobiose metabolism shown to induce diarrhea in rats (Moinuddin and Lee, 1958). In an attempt to moderate post-transplant diarrhea, practitioners regularly reduce dosages of the immunosuppressant mycophenolate mofetil, despite the increased risk of graft failure (Bunnapradist et al., 2006). By investigating how immunosuppression leads to differences in microbiome function, novel targets for prevention or treatment of post-transplant diarrhea might remove the requirement for reduction of the immunosuppressant dose.

There is emerging evidence for an association between transplantation-linked microbiome change and acute rejection of the organ. Disparity in the microbiome profiles of patients following non-rejection or acute rejection of transplanted organs has been observed in small bowel transplantation (Oh et al., 2012), and similar findings have been reported in a pilot study following kidney transplantation (Lee et al., 2014). Whether changes in the microbiome precede or follow acute rejection remains to be shown. Should they follow or non-causally precede acute rejection, microbial signifiers of rejection could provide a potential biomarker for early diagnosis (Fricke et al., 2014; Ren et al., 2014). If these changes precede acute rejection due to causality, they may instead provide a modifiable target for prevention. The composition of the microbiota, and the metabolites produced, can promote both inflammatory and tolerogenic immune responses towards transplanted organs (Ardalan and Vahed, 2017). Short-chain fatty acids produced by intestinal microbiota may provide protection against local and systemic inflammation, oxidative cellular stress, cell infiltration/activation, and apoptosis, as in murine models of acute kidney injury (Andrade-Oliveira et al., 2015). Identifying features causing such responses could, therefore, initiate the development of pre- or pro- biotic therapies aiming to improve long-term allograft outcome (Ardalan and Vahed, 2017).

## Could Delayed Kidney Function Leading to Gut Dysfunction Favor Rejection?

The structure of the gut microbiome is known to be altered in individuals with kidney disease (Al Khodor and Shatat, 2017; Nallu et al., 2017). Investigations in humans and rat models have shown differences between the gut microbiome in uremic subjects with ESRD and healthy controls (Vaziri et al., 2013a). The effect of renal transplantation has, however, not been extensively investigated. Renal dysfunction with increased serum urea leads to intestinal barrier dysfunction and disruption of the epithelial tight junction (Vaziri et al., 2013b). Such disruptions allow bacterial fragments and toxins to translocate from the gut microbiome into the bloodstream, promoting chronic systemic inflammation (Vaziri et al., 2012); whether this has a causal or exacerbating affect in co-morbidities associated with ESRD is unclear. After transplantation, there is some degree in variability as to how quickly the kidney allograft begins to function, although in recent years the incidence of delayed graft function has increased, possibly due to the use of expanded donor criteria, to within the range of 20–45% of cases (Yarlagadda et al., 2009; Matas et al., 2014; Willicombe et al., 2017; Jansen et al., 2018). Delayed graft function may expose the patient to a longer period of uremia and an increased risk of gut dysfunction, systemic inflammation, and allograft rejection. A delayed graft function of more than six days has been found to strongly decrease the long-term survival of transplanted kidneys (Giral-Classe et al., 1998).

## Urinary Microbiome May Hold Key to Early Rejection Detection

Since the recognition of its medical relevance, the urinary microbiome has been receiving growing attention (Fouts et al., 2012; Wolfe et al., 2012; Aragon et al., 2018). The most frequently reported genera are *Lactobacillus* and *Streptococcus*, with *Alloscardovia*, *Burkholderia, Jonquetella, Klebsiella, Saccharofermentans, Rhodanobacter*, and *Veillonella* also found less frequently (Aragon et al., 2018). Whilst the importance of the urinary microbiome in health is still emerging, evidence from several studies confirm its composition is altered by some post-transplant situations (**Table 1**). A study comparing the urinary microbiome of 21 kidney transplant recipients with that of 8 healthy controls reported marked differences between the two groups (Rani et al., 2017). Under the multiple stressors of kidney transplantation (including antibiotics, immunosuppression, and environmental changes) the urinary microbiota of kidney-transplant recipients suggested an overall decrease in diversity when compared to healthy controls, alongside an increased abundance of opportunistic pathogens (*Escherichia coli* and *Enterococcus faecalis*) and may select for promotion of antibiotic resistance. The effect of elevated urinary urea concentrations on urinary tract infections caused by urealytic pathogens also warrants further investigation. In the future, frequent, longitudinal sampling of the patient’s urinary microbiome might be implemented to detect deviations from microbiome stability. If these changes are shown to precede organ damage or loss, this may be useful as a non-invasive method of early detection.

## Conclusion and Perspectives

**Figure 1:**
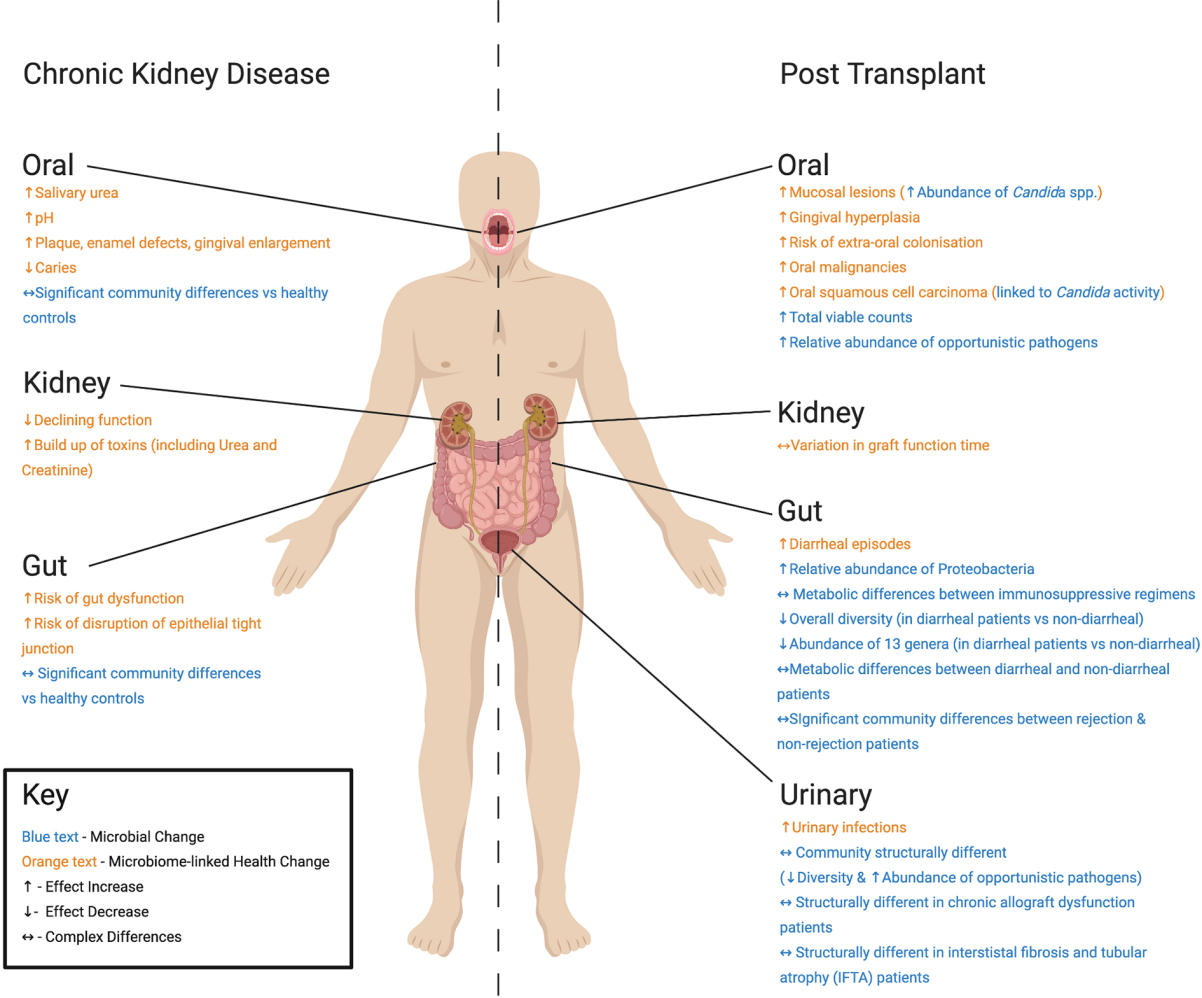
Summary of differences found at key body sites in studies of Chronic Kidney Disease (CKD) patients and kidney transplant recipients post-operation discussed in this review. For CKD patients, health changes associated with microbiome alteration include: (Oral) increased urea and pH (Lasisi et al., 2016), plaque, enamel defects, gingival enlargement and decreased caries (Al Nowaiser et al., 2003), (Kidney) declining function and build-up of toxins (Pandya et al., 2016), and (Gut) gut dysfunction and disruption of epithelial tight junction (Vaziri et al., 2013b). Reported microbial change includes significant changes in oral and gut communities compared with healthy controls (Hu et al., 2018; Hobby et al., 2019). For kidney transplant recipients, post-transplant health changes associated with microbiome alteration include: (Oral) increased mucosal lesions, gingival hyperplasia, risk of extra-oral colonization, and squamous cell carcinoma (Spolidorio et al., 2006), (Kidney) variations in time taken for graft to function (Yarlagadda et al., 2009; Willicombe et al., 2017), (Gut) increased diarrheal episodes (Lee et al., 2014), and (Urinary Tract) increases in urinary tract infections (Giessing, 2012). Microbial changes include: (Oral) increased abundance of *Candida* species (Spolidorio et al., 2006), total viable microorganism counts (Saraiva et al., 2006), and relative abundance of opportunistic pathogens (Diaz et al., 2013), (Gut) increased relative abundance of Proteobacteria (Lee et al., 2014), changes in microbial metabolism between (i) immunosuppressive regimens (Zaza et al., 2017), as well as microbial community structure in (ii) rejection and non-rejection patients (Lee et al., 2014) and (iii) diarrheal and non-diarrheal patients (Lee et al., 2019). (Urinary Tract) Structurally different microbiomes are also seen in transplant recipients (Fricke et al., 2014), as well as chronic allograft dysfunction (Wu et al., 2018) and interstistal fibrosis and tubular atrophy patients (Modena et al., 2017).

Alterations in the composition and activities of the human microbiome can have a range of consequences. Microbiome changes due to reduced kidney function in CKD and ESRD may be exacerbated during transplantation, with associated immunosuppression and restoration of kidney function. Whilst progress has been made in defining associations between the microbiome and kidney transplantation (summarized in **Figure 1**), the causal links and health consequences of these associations are not completely understood. Whilst few studies have investigated changes over prolonged timeframes in prospective cohorts, some pioneering studies have proposed how the microbiome alteration might translate to functional changes and alter post-transplant outcomes. An important goal of future research will be to tackle the challenges that kidney transplantation still faces. Indeed, evidence presented here implies a role for microbiome research in earlier detection and prevention of post-transplant infection and acute rejection, and achieving optimum individualized immunosuppression regimes to alleviate side effects. By increased understanding of how the microbiome and the immune system are affected by transplantation, clinically relevant information may be provided to enable treatment optimization for renal transplant recipients. To do so, large scale observations of kidney transplant recipients and donors are recommended to model the effects of co-occurring factors such as urea change, immunosuppression, and antibiotic administration. Translating these effects to animal models and *in vitro* systems, their relative impacts and interactions with microbial communities could then be isolated, understood, and, where required, interventions may be developed to alleviate co-morbidity, rejection and infection.

## Author Contributions

All authors ideated the review. PMC wrote the manuscript. All authors contributed to the article and approved the submitted version.

## Funding

This review was written as part of a PhD studentship (Project Reference: 2102580) funded by the Medical Research Council.

## Conflict of Interest

The authors declare that the research was conducted in the absence of any commercial or financial relationships that could be construed as a potential conflict of interest.
